# Physicochemical Composition of Local and Imported Honeys Associated with Quality Standards

**DOI:** 10.3390/foods12112181

**Published:** 2023-05-29

**Authors:** Hael S. A. Raweh, Ahmed Yacine Badjah-Hadj-Ahmed, Javaid Iqbal, Abdulaziz S. Alqarni

**Affiliations:** 1 Melittology Research Lab, Department of Plant Protection, College of Food and Agriculture Sciences, King Saud University, Riyadh 11451, Saudi Arabia; hraweh@ksu.edu.sa (H.S.A.R.); jiqbal@ksu.edu.sa (J.I.); 2 Department of Chemistry, College of Science, King Saud University, Riyadh 11451, Saudi Arabia; ybadjah@ksu.edu.sa

**Keywords:** honey, physicochemical properties, melissopalynological analysis, quality control

## Abstract

The compliance with honey standards is crucial for its validity and quality. The present study evaluated the botanical origin (pollen analysis) and physicochemical properties: moisture, color, electrical conductivity (EC), free acidity (FA), pH, diastase activity, hydroxymethylfurfural (HMF), and individual sugar content of forty local and imported honey samples. The local honey exhibited low moisture and HMF (14.9% and 3.8 mg/kg, respectively) than imported honey (17.2% and 23 mg/kg, respectively). Furthermore, the local honey showed higher EC and diastase activity (1.19 mS/cm and 11.9 DN, respectively) compared to imported honey (0.35 mS/cm and 7.6 DN, respectively). The mean FA of local honey (61 meq/kg) was significantly naturally higher than that of imported honey (18 meq/kg). All local nectar honey that originated from *Acacia* spp. exhibited naturally higher FA values that exceeded the standard limit (≤50 meq/kg). The Pfund color scale ranged from 20 to 150 mm in local honey and from 10 to 116 mm in imported honey. The local honey was darker, with a mean value of 102.3 mm, and was significantly different from imported honey (72.7 mm). The mean pH values of local and imported honey were 5.0 and 4.5, respectively. Furthermore, the local honey was more diverse in pollen grain taxa compared to imported honey. Local and imported honey elicited a significant difference regarding their sugar content within individual honey type. The mean content of fructose, glucose, sucrose, and reducing sugar of local honey (39.7%, 31.5%, 2.8%, and 71.2%, respectively) and imported honey (39.2%, 31.8%, 0.7%, and 72.0%, respectively) were within the permitted quality standards. This study indicates the necessity of increasing the awareness regarding quality investigations for healthy honey with good nutritional value.

## 1. Introduction

Honey is a natural substance produced by honey bees, and it originates from floral nectar and some plants exudates. Honey composition and quality characteristics are variable and are mainly affected by different factors such as soil composition, nectar source, climatic conditions, beekeeping practices, processing type, and storage conditions. Floral origin is quite influential on the physicochemical properties of honey such as electrical conductivity, color, moisture, pH, mineral content, and acidity level; conversely, other parameters, e.g., hydroxymethylfurfural (HMF) content and purity, are related to the manufacturing process [1]. The main constitutes of honey are carbohydrates (80–85%) and water (15–17%). Fructose and glucose are the most dominant sugars responsible for the majority of the physical and nutritional properties of honey. Small quantities of other sugars (disaccharides, trisaccharides, and oligosaccharides) are also present in honey, in addition to minerals, free amino acids, flavonoids, vitamins, enzymes, and phenolic and organic acids. The acidity level indicates the maturity of honey and characterizes its stability and changes of quality during storage [2]. There is an increasing interest in alternative medicine for public health, and honey is a main element in this regard. Therefore, honey consumption rates are increasing in Saudi Arabia [3]. Moreover, limited production of honey and inappropriate agricultural practices have led to an increase in honey adulteration [4]. Honey becomes an easy target of adulteration due to the high demand for its therapeutic and healing properties. Adulteration of honey could be direct by adding a substance to the honey such as cane sugar, beet sugar, and molasses, or indirect by feeding honeybee colonies with adulterating substances. Excessive heat used for pasteurization and packing, on the other hand, can have negative effects on honey quality, such as loss of enzyme activity [5]. Honey fermentation and spoilage may occur when honey is harvested with high humidity [6]. However, authenticity of honey is not only restricted to adulteration; indeed, post-harvest quality alterations are also possible during the flow season or in the store. In the recent past, various tools were developed to assess the quality and authenticity of honey as desired by the consumers, as well as to provide fair competition to honey producers. The international standards of honey quality parameters are available in different standards such as the Codex Alimentarius Standard and European Honey Directive [7]. In recent years, the importance of the physicochemical properties of honey has been increased, because these parameters are vital for issuing honey quality certificates [8]. Honey quality is generally accessed quantitatively by analyzing its composition, as described in international standards and legislations of honey (sucrose content ≤ 5%, fructose 31–42%, glucose 23–32%, reducing sugar ≥ 60%, moisture content ≤ 21%, water-insoluble content ≤ 0.1%, electrical conductivity ≤ 0.8 mS/cm, mineral content (ash) ≤ 0.6%, free acidity ≤ 50 meq/kg, diastase activity ≥ 8 DN (Schade units), and hydroxymethylfurfural (HMF) content ≤ 40 mg/kg) [7,9]. These specifications of honey were also adopted in Saudi Arabia by the Gulf Standardization Organization [10]. Honey is of special importance due to religious and cultural reasons in many Muslim countries, including Saudi Arabia. It is not only used as a sweetening additive but also as a healing agent. On the basis of official statistics, about 27,347 tons of honey were brought to the Saudi market last year (2021), of which, 3233 tons were produced locally and 24,114 tons were imported from different countries. In Saudi Arabia, the import of honey has increased during the last five years (2016–2020), from 13,568 to 16,441, 17,099, 18,526, and 24,114 tons, respectively [11]. Nevertheless, most of the locally produced honey is processed and marketed without a verified quality check and assessment of its origin information. This has led to increased honey adulteration and its marketing without verified quality. This study is of particular importance in order to add a comprehensive database of characterizing Saudi Arabian honey, as well as imported honey, which may contribute, if available, positively in terms of reformulating a proper and nationally accepted honey quality standard. Thus, the current study investigated the different physicochemical attributes of honey composition associated with quality standards from honey of local and imported origins.

## 2. Materials and Methods

The botanical origin (pollen analysis) and physicochemical characteristics such as moisture content, color, electrical conductivity (EC), free acidity (FA), pH, hydroxymethylfurfural (HMF), sugar content, and diastase activity (DN) of local and imported honey samples were evaluated according to the recommended methods [12,13,14,15,16,17,18,19,20,21,22]. The honey analyses were performed at the Honey Quality Research Laboratory, Department of Plant Protection, King Saud University, Riyadh.

### 2.1. Honey Samples

Twenty samples of each honey type (local and imported) of diversified botanical origin were collected from different sources in Saudi Arabia during 2020–2021 (Table 1). The honey samples were kept in the dark at room temperature until the subsequent analysis.

### 2.2. Melissopalynological Analysis

The pollen presence is fundamental for the melissopalynological analysis of honey [9]. The presence of pollen in the honey samples and the botanical origins of honey samples was tested according to the recommended protocols [15,16,17]. Briefly, ten grams of honey was mixed in 20 mL of warm distilled water (40 °C), centrifuged for 10 min at 2500 rpm, poured into a small tube, and centrifuged again for 10 min. The entire sediment was put on a slide, spread out over an area of 20 mm^2^, and dried by slight heating at 40 °C. The sediment was mounted with glycerin gelatin and liquefied by heating in a water bath at 40 °C [18]. The identification of pollen grain in the treated honey samples was performed according to the pollen atlas [19].

### 2.3. Physicochemical Analysis

The color, moisture content, EC, FA, pH, HMF, DN, and sugar content of local and imported honey samples were determined as per the recommended protocol [15]. Every honey sample was tested three times for every parameter, and the data were expressed as mean values.

#### 2.3.1. Color Analysis

The Pfund scale was used to measure the color intensity of honey samples according to the recommended protocol [15,21]. Half of the cuvette was filled with homogenous honey (without air bubbles) using a 10 mm light path. Color grades (0–150 mm) were determined using a color photometer (HI 96785, Hanna^®^ Instruments, Nusfalau, Romania), in which the cuvette was inserted. The analytical-grade glycerol standard was used to compare the Pfund grades of honey according to the United States Department of Agriculture [15,21].

#### 2.3.2. Moisture Content

The refractometric method [12,15] was used to measure the moisture content in terms of refractive indices with the help of a refractometer (Hammann^®^ honey refractometer, Hassloch, Germany) at ambient temperature. The refractive index directly increased with increases in the solid content of the honey sample. A drop of thoroughly mixed honey was put on the lens, and the lid of the refractometer was carefully closed for the even spreading of honey without any air bubbles. The refractometer was held towards the light to record the interface position. Before the testing of every honey sample, the instrument was thoroughly cleaned and dried.

#### 2.3.3. Electrical Conductivity (EC)

The EC was measured using an EC meter (Hanna^®^ pH PPM Meter HI-9813-6N, Nusfalau, Romania). It was first calibrated with deionized water, and the conductance cell was dipped into 10% honey solution (10.0%). The reading of EC was recorded after the stabilization of the instrument [12,15].

#### 2.3.4. pH

Ten grams of honey was mixed in 75 mL deionized water. Honey solution was transferred into a beaker, and a pH meter (Hanna^®^ pH PPM Meter HI-9813-6N, Nusfalau, Romania) was put in the solution. The stable readings of pH were recorded from the pH meter [12,15].

#### 2.3.5. Free Acidity (FA)

FA was measured using the titrimetric method. Ten grams of honey was dissolved in 75 mL of deionized water. The honey solution was titrated with sodium hydroxide (NaOH 0.05 N) until the pH value reached at 8.5. The final acidity number was expressed in meq/kg [12,15].

#### 2.3.6. Hydroxymethylfurfural (HMF)

HMF was recorded by determining the absorbance of the solutions at 284 and 336 nm, which was done using a GenesysTM10S UV-visible spectrometer (Thermo Fisher Scientific, Shanghai, China) [18]. The following equation was used to calculate the HMF content:

HMF(mg/kg)=(A284)−(A336)×149.7

where A284: absorbance value at 284 nm, A336: absorbance at 336 nm, and 149.7: a factor calculated by the molecular weight of HMF and the mass of the sample [12,15].

#### 2.3.7. Diastase Activity (DN)

The diastase number (DN) displaying the diastase activity and the DN of the honey samples was measured using the recommended protocol [7,15,18,22]. The absorbance of samples was recorded, and a calibration curve was formulated.

#### 2.3.8. Sugar Content

The percentages of sugar contents (fructose, glucose, sucrose, and reducing sugar) in honey samples were measured using high-performance liquid chromatography HPLC (Agilent Technologies^®^, Santa Clara, CA, USA) with RID detector and carbohydrate column). Sample preparation for HPLC was performed according to Raweh et al. [18]. The chromatogram peaks of the sugars were identified by comparison with those of previously injected standard sugars [15,20].

### 2.4. Statistical Analysis

The data of different physicochemical properties of honey are expressed as mean ± SE. The quantified variables of the honey samples were compared using the analysis of variance (ANOVA) and Duncan’s multiple range test. The statistical significance (*p* < 0.05) for the parameter values was calculated using SAS^®^ 9.2 software.

## 3. Results

### 3.1. Presence of Pollen Grains

The melissopalynological studies revealed the presence of different types of pollen grains in all tested local and imported honey samples that originated from diverse topographical origins (Table 2). A great diversity of pollens was observed in the pollen spectra. The majority of pollens belonged to four families (Fabaceae, Asteraceae, Rhamnaceae, and Capparaceae), which were detected from local honey samples. In imported honey samples, the majority of pollens that belonged to three families (Fabaceae, Asteraceae, and Rhamnaceae) were detected, but the pollen diversity was lower than those of local honey (Table 2).

### 3.2. Physicochemical Analysis of Honey

The physicochemical properties (moisture content, color, EC, pH, FA, HMF, DN, and sugar contents) were determined from the local and imported honey samples. The majority of the local and imported honey exhibited adequate quality physicochemical properties that were compatible with international regulation of honey quality [9,10]. Local honey samples showed certain physicochemical properties (Table 3 and Table 4) that were marked as relatively better (low moisture and HMF; high EC and DN) than that of imported honey samples, with a few exceptions. The low moisture content depicts the maturation of honey without any fermentation and long shelf life, low HMF with high DN illustrates the freshness and proper handling, and higher EC in the local exceptional nectar honey shows the presence of more mineral elements due to their botanical origin.

#### 3.2.1. Color

The honey color is dependent on their botanical origins, and it was significantly different among local honey (Table 3) and imported honey (Table 4) samples. The color ranged from white to dark amber for local honey, and extra white to dark amber for the imported honey. The Pfund scale ranged from 20 to 150 mm and 10 to 116 mm in local and imported honey, respectively (Table 3 and Table 4). The mean value for the Pfund color (102.3 ± 5.1) of local honey was significantly different from the imported honey (72.7 ± 3.59) (Table 5). The Pfund color scale of local and imported honey was within the suggested range of the International Codex [9,10,23].

#### 3.2.2. Moisture Content

The moisture content (%) was significantly different within the individual honey type, namely, local and imported honey samples (Table 3 and Table 4). The moisture content ranged from 13.1 ± 0.0 to 17.1 ± 0.1%, with a mean value of 14.9 ± 0.2% among local honey, (Table 3), and from 14.7 ± 0.0 to 19.4 ± 0.0%, with a mean value of 17.2 ± 0.3% for imported honey (Table 4).

Table 5 shows the moisture content values after comparison among local and imported honey samples. The imported honey possessed a significantly higher moisture content (17.2 ± 0.3%) than local honey (14.9 ± 0.2%) (Table 5), but these were within the permitted range (>20%) according to the international standards for honey [9,10].

#### 3.2.3. Electrical Conductivity (EC)

The values of EC ranged from 0.23 ± 0.0 to 2.00 ± 0.0 mS/cm, with a mean value of 1.19 ± 0.1 mS/cm (Table 3) for the local honey samples and from 0.05 ± 0.0 to 0.88 ± 0.0 mS/cm, with a mean value of 0.35 ± 0.1 mS/cm for the imported honey samples (Table 4). The mean EC value of local honey (1.19 ± 0.1 mS/cm) was significantly higher than that of imported honey (0.35 ± 0.1 mS/cm) (Table 5), which showed values within the permitted range (≤0.8 mS/cm) of international standards for blossom honey [9,10].

#### 3.2.4. pH

The pH values of the local honey (Table 3) and imported honey (Table 4) samples were acidic, and within the standard limit (3.4–6.1) of the international standard [9]. The pH values ranged from 3.5 ± 0.0 to 7.1 ± 0.0, with a mean of 5.0 ± 0.2 for local honey samples (Table 3), and 3.8 ± 0.0 to 6.1 ± 0.0, with a mean value of 4.5 ± 0.2 for imported honey samples (Table 4). Three local Sidr (*Ziziphus* spp.) honey samples (SDS4, SDS5, and SDS7) had pH values of 6.4 ± 0.0, 7.1 ± 0.0, and 6.6 ± 0.0, respectively (Table 3), which exceeded the standard limit (3.4–6.1). There was a significant difference within the samples of local (Table 3) and imported honey (Table 4). The mean pH values of local and imported honey did not show any significant differences (Table 5).

#### 3.2.5. Free Acidity (FA)

The results indicated a significant difference for FA level among honey samples of individual honey types (Table 3 and Table 4). The FA value ranged from 11 ± 0.0 to 110 ± 0.0 meq/kg (mean = 61 ± 8.3 meq/kg) for local honey samples (Table 3). The local honey samples (ACS1–7: originated from *Acacia* spp. plant), SDS1, SES, and SMS1–3 were characterized with high FA that exceeded the permitted limit (≤50 meq/kg) of honey standards (Table 3). The FA value for imported honey samples was within the permitted limit and ranged from 7 ± 0.0 to 37 ± 0.0 meq/kg (mean = 18 ± 2.1 meq/kg) (Table 4). The mean FA value (61 ± 8.3 meq/kg) of local honey was significantly higher as compared to the FA value (18 ± 2.1 meq/kg) of imported honey (Table 5).

#### 3.2.6. Hydroxymethylfurfural (HMF)

The HMF values of local honey (Table 3) and imported honey (Table 4) samples were lower than the standard limit (≤40 mg/kg) provided in the international standards [9] GSO [10]. The HMF values were significantly different among samples of individual honey type (Table 3 and Table 4). The HMF value of local honey ranged from 0.0 ± 0.0 to 29.2 ± 1.7 mg/kg (mean = 3.8 ± 1.5 mg/kg). The HMF value of imported honey ranged from 2.0 ± 0.0 mg/kg to 85 ± 2.1 mg/kg (mean = 23.0 ± 5.0 mg/kg) (Table 3 and Table 5). Three imported honey samples (PAS2, PAS4, and PAS6) had exceptional HMF values (67 ± 0.0, 42 ± 0.6, and 85 ± 2.1, respectively) (Table 4) that exceeded the standard limit (≤40 mg/kg). The mean HMF value was significantly lower in local honey samples (3.8 ± 1.5 mg/kg) than that of imported honey samples (23 ± 5.0 mg/kg) (Table 5).

#### 3.2.7. Diastase Activity (DN)

The diastase activity of honey is an important feature that is closely associated with the freshness of honey. The data of diastase activity were measured in terms of diastase number (DN). The values of local honey ranged from 5.2 ± 0.0 to 29.0 ± 0.0 DN, with mean of 11.9 ± 1.4 DN (Table 3), and were within the Codex standard limits (≥8). Out of twenty local honey samples, only two samples (SMS2 and SMS3) showed DN lower than the Codex standard limit (Table 3). The values of imported honey ranged from 0.0 ± 0.0 to 29.0 ± 0.0 DN, with a mean of 7.6 ± 1.6 DN. Out of twenty imported honey samples, eight samples (SMF, PAS3, PAS5, BFG, CSD, BMF, FMF, and KSD) showed DN values within the Codex standard limits (≥8), and the rest of all the samples had DN lower than the Codex standard limits (Table 4). The mean values of local honey (11.9 ± 1.4 DN) were higher and were significantly different from that of the imported honey samples (7.6 ± 1.6 DN) (Table 5).

### 3.3. Sugar Content of Honey

**Figure 1:**
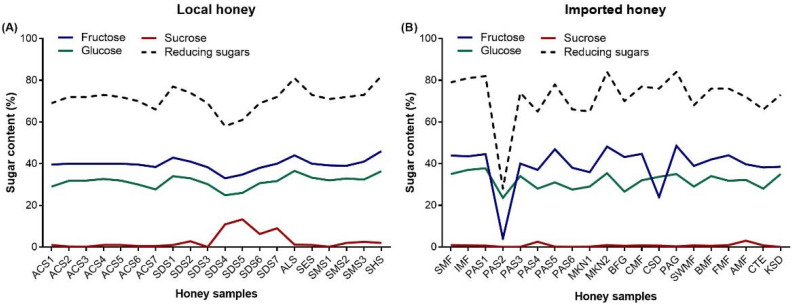
HPLC-based sugar profile of local honey and imported honey. (**A**) Local honey: ACS1–7 (*Acacia gerardii* honey from seven locations), SDS1–7 (Sidr, *Ziziphus* sp. honey from seven locations), ALS (alfalfa honey), SES (*Vachellia seyal* honey), SMS (*Acacia tortilis* honey), and SHS (Shafallah–caper bush honey, *Capparis spinose*). (**B**) Imported honey: SMF (multifloral honey, Spain), IMF (multifloral honey, India), PAS1–6 (honey imported from different countries but packed in KSA), MKN1–2 (manuka honey, New Zealand), BFG (black forest honey, Germany), CMF (multifloral honey, China), CSD (Sidr, *Ziziphus* sp. honey, China), PAG (*Robinia pseudoacacia* black locust honey, Germany), SWMF (multifloral honey, Switzerland), BMF (multifloral honey, United Kingdom), FMF (multifloral honey, France), AMF (multifloral honey, Australia), CTE (citrus honey, Egypt), and KSD (Sidr, *Ziziphus* sp. Pakistan).

The HPLC analysis revealed the percentage of sugar (fructose, glucose, and sucrose) detected in the tested local honey (Figure 1A) and imported honey samples (Figure 1B). The sequence pattern of sugar content was similar in local and imported honey samples, being within the permitted quality range (fructose: 31–42%, glucose: 23–32%, sucrose: ≤5%). The level of reducing sugar (fructose + glucose) was also within the permitted quality standard (≥60%) in both local and imported honey (Figure 1A,B).

The sugar content (fructose, glucose, sucrose, and reducing sugars) indicated significant differences within the individual type of local (Table 6) and imported honey (Table 7). Fructose and glucose were the two main carbohydrates that were detected in all the analyzed local and imported honey samples. Fructose percentage was relatively higher than that of glucose in local honey (Table 6) and imported honey, with an exception of two imported honey samples (PAS2 and CSD) that indicated a higher glucose percentage than that of fructose (Table 7).

In local honey (Table 6), the range of sugar content was 33 ± 0.0 to 46.0 ± 0.0% (mean: 39.7 ± 0.6%) for fructose, 24.9 ± 0.1 to 36.6 ± 0.0% (mean: 31.5 ± 0.7%) for glucose, 0.1 ± 0.0 to 13.3 ± 0.3% (mean: 2.8 ± 0.9%) for sucrose, and 58 ± 0.1 to 82 ± 0.2% (mean: 71.2 ± 1.3%) for reducing sugars. Four local honey samples (SDS4, SDS5, SDS6, and SDS7) showed sucrose contents that exceeded the permitted limits (≤5%) of International Codex and GSO standards [9,10].

In imported honey (Table 7), the range of sugar content was 4.0 ± 0.0 to 48.6 ± 0.1% (mean: 39.2 ± 2.3%) for fructose, 23.6 ± 0.0% to 37.8 ± 0.2 (mean: 31.8 ± 0.8%) for glucose, 0.0 ± 0.0% to 3.0 ± 0.0% (mean: 0.7 ± 0.2%) for sucrose, and 28 ± 0.0 to 84 ± 0.0% (mean: 72.0 ± 2.8%) for reducing sugars. All sugar contents were within the permitted limits (fructose, glucose, sucrose, and reducing sugar: 31–42%, 23–32%, ≤5%, and ≥60%, respectively) of the International Codex and GSO standard [9,10]. One local honey (SDS4: 58 ± 0.1%) (Table 6) and one imported honey (PAS2: 28 ± 0.0%) (Table 7) possessed lower percentages of reducing sugar than the permitted range (≥60%).

Table 8 revealed the mean values of various sugar contents in local and imported honey. Only the percentage of sucrose was significantly different among local (2.8%) and imported honey (0.7%). The contents of fructose, glucose, and reducing sugar were similar in both local and imported honey. The mean percentages of fructose, glucose, sucrose, and reducing sugar of the local and imported honey were within the permitted limits (fructose: 31–42%, glucose: 23–32%, sucrose: ≤5%, reducing sugar: ≥60%) of the International Codex and GSO standard [9,10].

## 4. Discussion

Pollen is a fundamental element in the analysis and quality evaluation of honey [24]. Melissopalynological analysis of honey provides the identification of the pollen types and potential plant source of honey [25]. This knowledge of pollen species is expedient in elucidating the sources of floral nectar that bees forage to produce honey of specific geographical and botanical sources [26]. In the present study, the pollen spectra from honey samples revealed that local honey had a relatively wide variety of botanical families than the imported honey. The possible explanation for the diversity in pollen content taxa between local and imported honey is because of different geographical regions, as well as the treatment of fine filtration. The local honey was without any fine filtration; unlike the imported honey, which might be commonly exposed to fine filtration to remove most of its pollen content before commercialization. According to USDA standards, commercial honey is filtered to remove suspended particles, including pollen grains [27]. Ponnuchamy et al. [28] reported the diversity of pollen spectra in the honey collected from one area at different times of the year.

We found that the color diversity among the honey samples (local and imported) ranged from white to dark amber, which is in accordance with the Pfund scale [21]. The diversity in honey color is common, and a previous study also reported the diversity ranged from colorless to amber and dark amber to black [14]. It is evident that the commercially available honey varied greatly in quality due to its color, flavor, and density over the globe [29]. Honey color is closely connected with botanical origin, and is an imperative to assess the honey quality [30]. Light-colored honey has a mild flavor, while dark honey has a more concentrated and rich flavor [31]. Furthermore, darker honey also has a high content of manganese, iron, phenolic compounds, and copper [14,32]. Many factors such as the environment, season, mineral, Maillard reaction, phenolic content, pollen, wax used, floral origin, and length of storage can affect the color of honey [25,32,33,34]. The Pfund color scale of local and imported honey was within the suggested range (0–150 mm) of the International Codex [9,10,23] and depended on the botanical origin.

The moisture content in the honey is important to determine honey quality, stability, resistance to spoilage, resistance to fermentation, and granulation during storage [25]. We found a relatively higher mean moisture content in imported honey than local honey, but both were within the acceptable limit (<20%) of international standards [9,10,23]. The prevalent subtropical climate conditions of high temperature and low humidity in Saudi Arabia could be the reason for the low moisture level in local honey. Moisture level is also vulnerable to geographical moisture conditions (temperature and humidity) during honey production, level of honey maturity in the hive, content of floral nectar, harvesting time, processing techniques, storage conditions, and apiary management [35,36,37]. The low moisture content would be an advantage for long storage with the prolonged shelf life of honey [25,38]. Other studies also found comparable findings of low moisture content in Saudi honey [24,39,40].

The level of EC is an important indicator of the quality of honey [41]. Our result showed that the EC value of local nectar honey exceeded the permitted limit (0.8 mS/cm) of international and Gulf standards [9,10]. The level of EC depends on the presence of mineral contents, storage time, floral origin, proteins, and organic acids in honey [14,40,42]. The higher level of these contents resulted in the higher EC, and vice versa [43]. EC is the most appropriate parameter for differentiating the geographical source and identification of flora of honey [41]. The level of EC showed great variation depending on the floral origin of honey [38]. The storage, floral sources, and color of honey also affect the EC values, as dark honey provides a higher EC than light-colored honey due to differences in the levels of minerals [43]. The local Saudi honey was exceptional nectar honey, which is characterized by naturally higher EC, and likewise, previous studies also presented higher EC in Saudi honey [25,40,43].

The pH value is linked with the number of organic acids present in the honey. It can also be influenced by various other factors such as the presence of inorganic ions, as well as extraction and storage conditions, which affect the structure, stability, and shelf life of honey, as well as the fermentation process [14,37]. In the present study, the mean pH values of local and imported honey (5.0 ± 0.2 and 4.5 ± 0.2, respectively) were within the permitted limit (3.4–6.1) of standards [9,10,23]. Generally, our results regarding pH values are in agreement with those described in the literature from different countries [38,39]. We also found that few samples of local Sidr honey (*Ziziphus sp*.) exhibited higher pH (>6.1) than standard limits (3.40 to 6.10), which is in line with previous studies where Sidr honey revealed high pH [40].

FA is a characteristic that originally depends on the floral source, geographical origin, and climatic conditions. We demonstrated that the mean FA (18 ± 2.1 meq/kg) of imported honey samples was within the permitted range (≤50 meq/kg) of standards) and was in agreement with previous studies [44]. Conversely, the mean FA of local honey samples (61 ± 8.3 meq/kg) was higher than that of imported honey and exceeded the permitted limits (≤50 meq/kg). The high mean FA value in local honey was due to the honey samples that originated from *Acacia* spp. plants, which had high FA because of the nature of floral source [45]. The high FA value exceeding the permitted standards in honey originated from *Acacia* plants were in agreement with previous studies conducted in different Gulf countries, such as Oman, Saudi Arabia, and Yemen [33,40,46]. Irrespective of geographical origin, *Acacia* honey has distinctive acidic properties. The nature of the *Acacia* nectar and the effect of the honey harvest season of hot summer, as well as high-salinity soils, could be the possible reason for the acidity of *Acacia* honey [47]. In honey, essential acid gluconic acid is produced by oxidation of glucose with an enzyme glucose oxidase, which makes honey slightly acidic [20]. Thus, the increase in FA may be due the presence of a high level of gluconic acid in *Acacia* flowers as a rich source of nectar [48]. The variations in FA among local and imported honey samples might be due to differences in geographical conditions; the presence of organic acids, particularly gluconic acid; inorganic ions (phosphate and chloride); floral sources; the fermentation process; and the bee species [33]. A high FA value in *Acacia* honey samples could therefore be a feature of honey related to the floral origin of honey.

HMF is one of the most important criteria to monitor the freshness of honey, beekeeping practices, honey exposure to high temperature, and storage conditions [49]. In fresh honey, the level of HMF is naturally in small quantities, but its concentration increases with storage duration and prolonged heating [50]. HMF is an indicator for poor storage conditions at high temperature [18]. Our result revealed that mean HMF content of the local honey samples (3.8 ± 1.5 mg/kg) was lower than that of the imported honey samples (23.0 ± 5.0 mg/kg), but both were within the permitted limits (≤40 or 80 mg/kg) of honey standards [9,10,23]. Only in a few imported honey samples did HMF exceed these standards. These high HMF values in the present results might have been due to storage time and honey exposure to heating [20]. The accepted level of HMF in honey differs among countries, i.e., being greater in hot tropical countries, and should not exceed 80 mg/kg, whereas in other countries, 40 mg/kg is the maximum accepted level [9,10,23]. Our results are in agreement with the findings of previous studies [24,51]. The production of HMF can be increased with the presence of simple sugars (glucose and fructose), many acids and minerals in honey, in addition to honey processing practices or long storage [52].

The diastase enzyme is a significant enzyme secreted by bees during the conversion of nectar into honey, in addition to its floral source. It is greatly affected by the floral origin, climate, poor storage, and exposure of honey to heating; the activity of the diastase enzyme indicates the freshness of honey [53]. The storage duration and honey exposed to heating can modify the diastase activity of honey [54,55]. Our results exhibited that the diastase activity (11.9 ± 1.4 DN) of local honey met the requirements of international and local standards (≥8), with the exception of only two samples with lower diastase activity than the standard limits. Comparable values for diastase activity have been reported for Ethiopian, Argentinian, and Omani honey [56]. The mean diastase activity of imported honey was 7.6 ± 1.6 DN, and the majority of samples were out of the standard limits of international and local standards [9,10,23]. These results indicated that imported samples were either older, stored in poor conditions, or exposed to heating [55] that degraded the enzyme and resulted in decreased diastase activity. The diastase activity values are in agreement with the findings of Mesallam and El-Shaarawy [51].

The level of sugar content in honey is an important for its quality assessment [24,25]. Our results revealed that fructose, glucose, and sucrose were the most important sugars found in the analyzed honey samples, and the levels of these sugars were significantly different among local and imported honey samples. These sugars were sourced from the floral nectar that bees forage and consume during honey production, and the floral source can be identified from sugar analysis [57]. The mean levels of reducing sugar in local (71.2 ± 1.3%) and imported honey (72 ± 2.8%) were within the permitted range (≥60) of honey standards [9,10,23], and these outcomes were in confirmation with previous findings [24,51]. In the present study, fructose was the main sugar in the honey samples compared to glucose and sucrose. The mean fructose level was 39.7 ± 0.6% in local honey and 39.2 ± 2.3% in imported honey, and they were within the permitted range (31–42%) of honey standards [9,10,23]. Only two samples of imported honey had less fructose but higher glucose, indicating that these samples were possibly adulterated [24,58]. The mean percentage of glucose in our data was 31.5 ± 0.7% in local honey and 31.8 ± 0.8% in imported honey, being within the permitted range (23–32%) of honey standards [9,10,23]. These values are in agreement with findings of previous studies [56].

In our data, the local honey had a higher sucrose percentage (2.8 ± 0.9%) than that of imported honey (0.7 ± 0.2%) and was within the permitted range (≤5%) of honey standards [9,10,23]. Tigistu et al. [33] also found high sucrose (2.54 ± 0.40%) in Ethiopian honey. The normal levels of sucrose in most samples indicate that these honey samples were highly matured [50]. Some Sidr honey samples in our data had a higher level of sucrose, which could be attributed to the fact that some beekeepers harvest their honey before the complete sealing of honeycombs. This early harvest is related to the two short peaks of honey flow during Sidr flowering season [45,59,60].

The adulteration of commercial honey is a continued concern worldwide. Generally, adulteration of honey involves the addition of different sugary syrups such as C3 and C4 sugars and certain oligosaccharides. The common source of C4 sugar is sugarcane and corn, with C3 sugar coming from rice and beetroot, while starch-based polysaccharides come from rice and corn [61,62]. Resin technology, a new kind of adulteration, is also being used to produce adulterated honey, hide its origin, and eliminate any trace of contamination and antibiotics. The FDA has notified that the honey going through resin technology should not be labelled as honey. Resin technology can eliminate/alter the chemical components of honey color, flavor, and aroma; pollen; antibiotics; and residues. It also helps the commercial companies to customize the color, aroma, and flavor of honey [63]. The modified sugar syrups are difficult to catch because they are designed not to be detected by the regular testing sugar methods. An advanced global standard specialized testing using nuclear magnetic resonance (NMR) is under debate, which might be needed in order to verity the quality of honey, as well as to analyze the presence of modified sugars in the honey. NMR is a powerful analytical tool that can detect the presence and structure of different substances in honey [64].

We proposed a recommendation for the implication of NMR testing at the country level for the export and import of honey in order to authenticate the honey. A thorough surveillance with solid custom regulations could help to alleviate the import of honey adulterated with common sugars. A regular inspection of honey processing units for sampling honey and testing with NMR is also recommended because there is a high probability that adulterated honey with C3, C4, polysaccharides, and fructose syrups could bypass the normal purity tests.

## 5. Conclusions

The quality of local and imported honey was determined by evaluating their physicochemical properties such moisture, color, electrical conductivity, pH, diastase activity, free acidity, sugar content, and HMF. The majority of these tested parameters of local and imported honey complied with the different quality standards. The local honey showed lower moisture content and HMF, as well as higher diastase enzyme activity and EC, than the imported honey. The free acidity level was higher than the quality standards in the local exceptional nectar honey. The pollen analysis identified different types of pollen present in the honey samples, as well as their plant sources of floral nectar. The characterization and estimation of the physicochemical parameters of local and imported honey is crucial in order to monitor the quality of honey, prepare certification marks for validity, produce high-quality honey in Saudi Arabia, and propose new standards that are based on the characteristics of Saudi honey.

## Figures and Tables

**Table 1:** Detail of tested honey samples.

Origin of Honey	No.	Sample Code	Detail of Honey Type and Location
Native honey (Kingdom of Saudi Arabia, KSA)	1	ACS1	*Acacia gerardii* honey (Huraymila: 25°12″ N, 46°10″ E)
	2	ACS2	*Acacia gerardii* honey (Hail: 27°31″ N, 41°41″ E)
	3	ACS3	*Acacia gerardii* honey (Riyadh: 24°24″ N 46°71″ E)
	4	ACS4	*Acacia gerardii* honey (Al Qassim: 25°49″ N, 42°51″ E)
	5	ACS5	*Acacia gerardii* honey (Al Taif: 21°16″ N, 44°25″ E)
	6	ACS6	*Acacia gerardii* honey (Asir: 18°13″ N, 42°23″ E)
	7	ACS7	*Acacia gerardii* honey (Al Ahsa: 25°17″ N, 49°29″ E)
	8	SDS1	Sidr, *Ziziphus* sp. honey (Huraymila: 25°12″ N, 46°10″ E)
	9	SDS2	Sidr, *Ziziphus* sp. honey (Hail: 27°31″ N, 41°41″ E)
	10	SDS3	Sidr, *Ziziphus* sp. honey (Riyadh: 24°24″ N 46°71″ E)
	11	SDS4	Sidr, *Ziziphus* sp. honey (Al Qassim: 25°49″ N, 42°51″ E)
	12	SDS5	Sidr, *Ziziphus* sp. honey (Al Taif: 21°16″ N, 44°25″ E)
	13	SDS6	Sidr, *Ziziphus* sp. honey (Asir: 18°13″ N, 42°23″ E)
	14	SDS7	Sidr, *Ziziphus* sp. honey (Al Ahsa: 25°17″ N, 49°29″ E)
	15	ALS	Alfalfa honey (Al Qassim: 25°49″ N, 42°51″ E)
	16	SES	*Vachellia seyal* honey (Huraymila: 25°12″ N, 46°10″ E)
	17	SMS1	*Acacia tortilis* honey (Huraymila: 25°12″ N, 46°10″ E)
	18	SMS2	*Acacia tortilis* honey (Riyadh: 24°24″ N 46°71″ E)
	19	SMS3	*Acacia tortilis* honey (Al Taif: 21°16″ N, 44°25″ E)
	20	SHS	Shafallah–caper bush honey, *Capparis spinose* (Al Taif: 21°16″ N, 44°25″ E)
Imported Honey (from different countries of the world)	1	SMF	Multifloral honey, Spain
	2	IMF	Multifloral honey, India
	3	PAS1	Honey imported from different countries but packed in KSA
	4	PAS2	Honey imported from different countries but packed in KSA
	5	PAS3	Honey imported from different countries but packed in KSA
	6	PAS4	Honey imported from different countries but packed in KSA
	7	PAS5	Honey imported from different countries but packed in KSA
	8	PAS6	Honey imported from different countries but packed in KSA
	9	MKN1	Manuka honey, New Zealand
	10	MKN2	Manuka honey, New Zealand
	11	BFG	Black forest honey, Germany
	12	CMF	Multifloral honey, China
	13	CSD	Sidr, *Ziziphus* sp. honey, China
	14	PAG	*Robinia pseudoacacia* Black locust honey, Germany
	15	SWMF	Multifloral honey, Switzerland
	16	BMF	Multifloral honey, United Kingdom
	17	FMF	Multifloral honey, France
	18	AMF	Multifloral honey, Australia
	19	CTE	Citrus honey, Egypt
	20	KSD	Sidr, *Ziziphus* sp. honey, Pakistan

**Table 2:** Microscopic analyses of the pollen grain types present in the local and imported honey samples.

Origin of Honey	No.	Sample Code	Detail of Pollen Grains
Native honey	1	ACS1	Fabaceae, and others.
	2	ACS2	Fabaceae, and others.
	3	ACS3	Fabaceae, Rhamnaceae, and others.
	4	ACS4	Fabaceae, Asteraceae, and others.
	5	ACS5	Fabaceae, Capparaceae, Malvaceae, Asteraceae.
	6	ACS6	Fabaceae, and others.
	7	ACS7	Fabaceae, Asteraceae, and others.
	8	SDS1	Rhamnaceae, and others.
	9	SDS2	Rhamnaceae, Fabaceae, Asteraceae, and others.
	10	SDS3	Rhamnaceae, Tamaricaceae, Capparaceae, Asteraceae, Combretaceae, and others.
	11	SDS4	Rhamnaceae, Fabaceae, Combretaceae, Capparaceae, and others.
	12	SDS5	Rhamnaceae, Capparaceae, Fabaceae, and others.
	13	SDS6	Rhamnaceae, Capparaceae, and others.
	14	SDS7	Rhamnaceae, Fabaceae, Combretaceae, Capparaceae, and others.
	15	ALS	Fabaceae, Capparaceae, and others.
	16	SES	Fabaceae, Tamaricaceae, Asteraceae, and Capparaceae.
	17	SMS1	Fabaceae, Asteraceae, and others.
	18	SMS2	Fabaceae, Rhamnaceae, Asteraceae, Capparaceae, and others.
	19	SMS3	Fabaceae, Capparaceae, and others.
	20	SHS	Capparaceae, Fabaceae, Rhamnaceae, Asteraceae, and others.
Imported honey	1	SMF	Malvaceae, Asteraceae, Fabaceae, Santalaceae, and others.
	2	IMF	Fabaceae, and others.
	3	PAS1	Fabaceae, and others.
	4	PAS2	Fabaceae, Asteraceae, and others.
	5	PAS3	Myrtaceae, Fabaceae, Asteraceae, and others.
	6	PAS4	Rhamnaceae, Fabaceae, Asteraceae, Malvaceae, Solanaceae, and others.
	7	PAS5	Fabaceae, Asteraceae, Malvaceae, and others.
	8	PAS6	Fabaceae, Asteraceae, and others.
	9	MKN1	Myrtaceae, Lamiaceae, and others.
	10	MKN2	Myrtaceae, Solanaceae, Asteraceae, and others.
	11	BFG	Rhamnaceae, and others.
	12	CMF	Rosaceae, and others.
	13	CSD	Rhamnaceae, Fabaceae, Rosaceae, and Asteraceae.
	14	PAG	Fabaceae, Convolvulaceae, and others.
	15	SWMF	Rhamnaceae, Fabaceae, Ericaceae, Fabaceae, and others.
	16	BMF	Asteraceae, and others.
	17	FMF	Pinaceae, and others.
	18	AMF	Rutaceae, and others.
	19	CTE	Rutaceae, Fabaceae, Rhamnaceae, Asteraceae, Solanaceae, and others.
	20	KSD	Rhamnaceae, Fabaceae, Asteraceae, and others.

Others: pollen families that were not identified.

**Table 3:** Physicochemical properties of local honey samples.

No.	Sample Code	Color	* Pfund Color (mm)	Moisture (%)	EC (mS/cm)	pH	Free Acidity (meq/kg)	HMF (mg/kg)	DN
1	ACS1	Dark amber	144 ± 0.0 b	13.8 ± 0.0 k	1.91 ± 0.0 b	5.2 ± 0.0 e	106 ± 0.3 b	1.6 ± 0.2 efg	8.7 ± 0.3 gh
2	ACS2	Amber	113 ± 0.0 g	14.3 ± 0.0 j	1.51 ± 0.0 e	4.5 ± 0.0 l	95 ± 0.0 e	1.6 ± 0.2 efg	10.0 ± 0.0 f
3	ACS3	Dark amber	150 ± 0.0 a	15.6 ± 0.0 c	1.35 ± 0.0 h	4.5 ± 0.0 l	100 ± 0.0 d	6.3 ± 0.1 c	11.0 ± 0.0 e
4	ACS4	Dark amber	125 ± 0.0 c	14.4 ± 0.0 j	1.55 ± 0.0 d	4.9 ± 0.0 h	90 ± 0.0 f	0.7 ± 0.3 fg	10.0 ± 0.0 f
5	ACS5	Dark amber	120 ± 0.0 d	14.4 ± 0.0 j	1.27 ± 0.0 i	4.5 ± 0.0 l	66 ± 0.0 i	10.9 ± 0.3 b	8.0 ± 0.0 i
6	ACS6	Dark amber	150 ± 0.0 a	14.5 ± 0.0 ij	1.83 ± 0.0 c	5.1 ± 0.0 f	105 ± 0.0 b	0.9 ± 0.2 fg	10.9 ± 0.1 e
7	ACS7	Dark amber	150 ± 0.0 a	13.1 ± 0.0 l	2.00 ± 0.0 a	4.8 ± 0.0 i	110 ± 0.0 a	0.2 ± 0.0 fg	10.9 ± 0.1 e
8	SDS1	Dark amber	115 ± 0.0 e	15.2 ± 0.0 d	1.43 ± 0.0 f	5.0 ± 0.0 g	102 ± 0.3 c	0.3 ± 0.3 fg	28.7 ± 0.3 a
9	SDS2	Extra light amber	44 ± 0.0 n	14.9 ± 0.0 fgh	0.62 ± 0.0 m	4.9 ± 0.0 h	15 ± 0.0 n	1.1 ± 0.2 fg	29.0 ± 0.0 a
10	SDS3	Light amber	60 ± 0.0 m	15.03 ± 0.0 def	0.55 ± 0.0 n	5.1 ± 0.0 f	15 ± 0.0 n	0.8 ± 0.1 fg	17.4 ± 0.0 b
11	SDS4	Amber	103 ± 0.0 i	15.1 ± 0.0 de	1.39 ± 0.0 g	6.4 ± 0.0 c	23 ± 0.0 l	1.3 ± 0.2 efg	11.0 ± 0.0 e
12	SDS5	Amber	86 ± 0.0 j	15.0 ± 0.0 efg	1.33 ± 0.0 h	7.1 ± 0.0 a	11 ± 0.0 o	0.7 ± 0.2 fg	12.0 ± 0.0 d
13	SDS6	Light amber	81 ± 0.0 k	15.6 ± 0.0 c	1.45 ± 0.0 f	5.4 ± 0.0 d	20 ± 0.0 m	0.0 ± 0.0 g	10.3 ± 0.0 f
14	SDS7	Light amber	75 ± 0.0 l	14.7 ± 0.1 hi	0.66 ± 0.0 l	6.6 ± 0.0 b	11 ± 0.0 o	3.4 ± 0.3 de	8.0 ± 0.0 i
15	ALS	White	31 ± 0.0 o	16.0 ± 0.0 b	0.23 ± 0.0 o	3.5 ± 0.0 n	30 ± 0.0 k	2.2 ± 0.4 efg	9.0 ± 0.0 g
16	SES	Amber	103 ± 0.0 i	13.8 ± 0.0 k	1.24 ± 0.0 i	4.6 ± 0.0 k	67 ± 0.0 h	2.4 ± 0.4 ef	8.3 ± 0.0 hi
17	SMS1	Amber	114 ± 0.0 f	16.0 ± 0.0 b	1.19 ± 0.0 j	4.6 ± 0.0 k	75 ± 0.0 g	5.6 ± 0.1 cd	9.0 ± 0.0 g
18	SMS2	Amber	112 ± 0.0 h	14.8 ± 0.1 gh	0.96 ± 0.0 k	4.6 ± 0.0 k	65 ± 0.0 j	29.2 ± 1.7 a	5.6 ± 0.0 j
19	SMS3	Dark amber	150 ± 0.0 a	15.0 ± 0.0 efg	1.19 ± 0.0 j	4.7 ± 0.0 j	90 ± 0.0 f	7.2 ± 0.1 c	5.2 ± 0.0 j
20	SHS	White	20 ± 0.0 p	17.1 ± 0.1 a	0.23 ± 0.0 o	3.8 ± 0.0 m	29 ± 0.3 k	0.2 ± 0.1 fg	15.3 ± 0.0 c

Means with the same letters are not significantly different from each other (*p* < 0.05, Tukey’s test). * Color was determined in mm on the Pfund scale according to the U.S. Department of Agriculture classifications (water white: <9, extra white: 9–17, white: 18–34, extra light amber: 35–50, light amber: 51–85, amber: 86–114, dark amber: >114). Codex Alimentarius Standard (moisture: ≤20%, Pfund color: 0–150 mm; EC: ≤0.8 mS/cm; pH: 3.4–6.1; FA: ≤50 meq/kg; HMF: ≤40 mg/kg (in tropical regions: 80 mg/kg); DN: ≥8) [9,10].

**Table 4:** Physicochemical properties of imported honey samples.

No.	Sample Code	Color	* Pfund Color	Moisture %	EC mS/cm	pH	Free Acidity (meq/kg)	HMF (mg/kg)	DN
1	SMF	Light amber	70 ± 0.0 k	17.2 ± 0.0 h	0.26 ± 0.0 j	4.0 ± 0.0 j	20 ± 0.0 d	20 ± 0.0 h	11.0 ± 0.0 d
2	IMF	Light amber	72 ± 0.0 j	17.5 ± 0.0 ef	0.13 ± 0.0 m	4.4 ± 0.0 g	14 ± 0.0 g	38 ± 0.0 d	0.0 ± 0.0 m
3	PAS1	Light amber	68 ± 0.0 i	17.7 ± 0.0 de	0.25 ± 0.0 j	4.1 ± 0.0 i	20 ± 0.0 d	40 ± 0.0 cd	0.0 ± 0.0 m
4	PAS2	Light amber	60 ± 0.0 m	16.8 ± 0.0 i	0.05 ± 0.0 o	4.0 ± 0.0 j	7 ± 0.0 l	67 ± 0.0 b	0.0 ± 0.0 m
5	PAS3	Amber	93 ± 0.0 f	14.7 ± 0.0 m	0.88 ± 0.0 a	7.2 ± 0.0 a	8 ± 0.0 k	2 ± 0.0 k	8.0 ± 0.0 f
6	PAS4	Light amber	85 ± 0.0 g	18.4 ± 0.0 c	0.10 ± 0.0 n	3.9 ± 0.0 k	11 ± 0.0 i	42 ± 0.6 c	6.6 ± 0.0 gh
7	PAS5	White	28 ± 0.0 q	17.9 ± 0.0 d	0.17 ± 0.0 l	4.5 ± 0.0 f	7 ± 0.0 l	24 ± 0.3 g	8.3 ± 0.0 ef
8	PAS6	Amber	109 ± 0.0 b	18.7 ± 0.1 b	0.30 ± 0.0 h	3.8 ± 0.0 l	27 ± 0.0 c	85 ± 2.1 a	5.0 ± 0.0 i
9	MKN1	Amber	99 ± 0.0 d	19.4 ± 0.0 a	0.48 ± 0.0 f	4.0 ± 0.0 j	30 ± 0.0 b	10 ± 0.0 j	7.0 ± 0.0 fg
10	MKN2	Extra light amber	50 ± 0.0 n	16.7 ± 0.1 i	0.59 ± 0.0 d	4.2 ± 0.0 h	30 ± 0.0 b	14 ± 0.1 i	2.5 ± 0.0 jk
11	BFG	Light amber	78 ± 0.0 i	15.8 ± 0.0 k	0.73 ± 0.0 c	4.5 ± 0.0 f	37 ± 0.0 a	2 ± 0.1 k	14.0 ± 0.0 c
12	CMF	White	33 ± 0.0 p	18.2 ± 0.0 c	0.28 ± 0.0 i	4.7 ± 0.0 d	10 ± 0.0 j	10 ± 0.2 j	2.0 ± 0.0 kl
13	CSD	Light amber	82 ± 0.0 h	16.9 ± 0.0 i	0.38 ± 0.0 g	5.3 ± 0.0 c	10 ± 0.0 j	2 ± 0.3 k	9.4 ± 0.0 e
14	PAG	Extra white	10 ± 0.0 r	17.46 ± 0.1 fg	0.16 ± 0.0 l	3.8 ± 0.0 l	15 ± 0.0 f	27 ± 0.0 f	5.5 ± 1.1 hi
15	SWMF	Amber	98 ± 0.0 e	17.3 ± 0.1 gh	0.39 ± 0.0 g	3.9 ± 0.0 k	30 ± 0.0 b	3 ± 0.0 k	7.5 ± 0.0 fg
16	BMF	Amber	100 ± 0.0 c	18.2 ± 0.1 c	0.06 ± 0.0 o	4.6 ± 0.0 e	7.0 ± 0.0 l	9 ± 0.0 j	20.0 ± 0.0 b
17	FMF	Light amber	82 ± 0.0 h	17.4 ± 0.0 fgh	0.54 ± 0.0 e	4.4 ± 0.0 g	30 ± 0.0 b	9 ± 0.1 j	10.9 ± 0.0 d
18	AMF	Extra light amber	43 ± 0.0 o	16.2 ± 0.0 j	0.25 ± 0.0 j	4.7 ± 0.0 d	10 ± 0.0 j	20 ± 0.7 h	3.6 ± 0.0 j
19	CTE	Light amber	78 ± 0.0 i	14.9 ± 0.0 l	0.20 ± 0.0 k	3.9 ± 0.0 k	16 ± 0.0 e	32 ± 1.3 e	0.7 ± 0.0 lm
20	KSD	Drak amber	116 ± 0.0 a	16.9 ± 0.0 i	0.84 ± 0.0 b	6.1 ± 0.0 b	13 ± 0.3 h	3 ± 0.0 k	29.0 ± 0.0 a

Means with the same letters are not significantly different from each other (*p* < 0.05, Tukey’s test). * Color was determined in mm on the Pfund scale according to the U.S. Department of Agriculture classifications (water white: <9, extra white: 9–17, white: 18–34, extra light amber: 35–50, light amber: 51–85, amber: 86–114, dark amber: >114). Codex standard (moisture: ≤20%, Pfund color: 0–150 mm; EC: ≤0.8 mS/cm; pH: 3.4–6.1; FA: ≤50 meq/kg; HMF: ≤40 mg/kg (in tropical regions: 80 mg/kg); DN: ≥8) [9,10].

**Table 5:** Comparison among mean values of tested physicochemical properties of local and imported honey.

Physicochemical Properties	Local Honey (Mean ± SE)	Imported Honey (Mean ± SE)	*p*-Value
Moisture (%)	14.9 ± 0.2	17.2 ± 0.3	0.018 *
Color	102.3 ± 5.1	72.7 ± 3.59	0.004 *
EC (mS/cm)	1.19 ± 0.1	0.35 ± 0.1	<0.000 *
pH	5.0 ± 0.2	4.5 ± 0.2	0.424
FA (meq/kg)	61 ± 8.3	18 ± 2.1	<0.000 *
HMF (mg/kg)	3.8 ± 1.5	23 ± 5.0	<0.000 *
DN	11.9 ± 1.4	7.6 ± 1.6	0.040 *

Asterisk represents a significant difference between local and imported honey for each single attribute (*p* < 0.05, *t*-test). Codex Alimentarius Standard (moisture: ≤20%, Pfund color: 0–150 mm; EC: ≤0.8 mS/cm; pH: 3.4–6.1; FA: ≤50 meq/kg; HMF: ≤40 mg/kg (in tropical regions: 80 mg/kg); DN: ≥8) [9,10].

**Table 6:** Analysis of sugar content (%) present in local honey samples.

No.	Sample Code	Sugar Content (%)			
		Fructose	Glucose	Sucrose	Reducing Sugars
1	ACS1	39.6 ± 0.0 ef	29.0 ± 0.0 h	1.0 ± 0.0 gh	69 ± 0.0 ef
2	ACS2	40.0 ± 0.0 e	31.8 ± 0.2 f	0.3 ± 0.0 i	72 ± 0.2 cde
3	ACS3	40.0 ± 0.0 e	31.9 ± 0.0 f	0.2 ± 0.0 i	72 ± 0.0 cde
4	ACS4	40.0 ± 0.0 e	32.7 ± 0.0 cde	1.0 ± 0.0 gh	73 ± 0.0 cd
5	ACS5	40.0 ± 0.0 e	31.9 ± 0.1 ef	1.0 ± 0.0 gh	72 ± 0.1 cde
6	ACS6	39.6 ± 0.0 ef	30.0 ± 0.0 g	0.5 ± 0.0 hi	70 ± 0.0 def
7	ACS7	38.4 ± 0.2 gh	27.7 ± 0.1 i	0.5 ± 0.0 hi	66 ± 0.2 f
8	SDS1	38.3 ± 0.4 h	30.2 ± 0.3 g	0.1 ± 0.0 i	69 ± 0.8 ef
9	SDS2	42.9 ± 0.1 c	34.0 ± 0.0 b	1.0 ± 0.0 gh	77 ± 0.1 b
10	SDS3	41.0 ± 0.0 d	33.0 ± 0.0 cd	2.8 ± 0.0 e	74 ± 0.0 bc
11	SDS4	33.0 ± 0.0 j	24.9 ± 0.1 k	10.9 ± 0.1 b	58 ± 0.1 g
12	SDS5	34.8 ± 0.1 i	26.0 ± 0.0 j	13.3 ± 0.3 a	61 ± 0.0 g
13	SDS6	38.0 ± 0.0 h	30.7 ± 0.3 g	6.3 ± 0.3 d	69 ± 0.3 ef
14	SDS7	40.0 ± 0.0 e	31.7 ± 0.1 f	9.0 ± 0.0 c	72 ± 0.1 cde
15	ALS	44.0 ± 0.0 b	36.6 ± 0.0 a	1.2 ± 0.2 g	81 ± 0.0 a
16	SES	40.0 ± 0.0 e	33.3 ± 0.3 bc	1.0 ± 0.0 gh	73 ± 0.3 bcd
17	SMS1	39.2 ± 0.2 f	32.0 ± 0.0 ef	0.2 ± 0.0 i	71 ± 0.2 cde
18	SMS2	39.0 ± 0.0 fg	32.9 ± 0.0 cd	2.0 ± 0.0 f	72 ± 0.1 cde
19	SMS3	41.0 ± 0.0 d	32.4 ± 0.2 def	2.5 ± 0.0 ef	73 ± 0.2 bc
20	SHS	46.0 ± 0.0 a	36.4 ± 0.2 a	2.0 ± 0.0 f	82 ± 0.2 a
Mean		39.7 ± 0.6	31.5 ± 0.7	2.8 ± 0.9	71.2 ± 1.3

Means with the same letters are not significantly different from each other (*p* < 0.05, Tukey’s test). Codex Alimentarius Standard (fructose: 31–42%, glucose: 23–32%, sucrose: ≤5%, reducing sugar: ≥60%) [9,10].

**Table 7:** Analysis of sugar content (%) present in imported honey samples.

No.	Sample Code	Sugar Content (%)			
		Fructose	Glucose	Sucrose	Reducing Sugars
1	SMF	43.9 ± 0.0 abc	35.0 ± 0.0 c	0.9 ± 0.0 c	79 ± 0.0 abc
2	IMF	43.5 ± 0.0 abc	37.0 ± 0.0 b	0.8 ± 0.0 cd	81 ± 0.0 abc
3	PAS1	44.6 ± 0.4 abc	37.8 ± 0.2 a	0.7 ± 0.0 cd	82 ± 0.5 ab
4	PAS2	4.0 ± 0.0 e	23.6 ± 0.0 j	0.0 ± 0.0 f	28 ± 0.0 j
5	PAS3	40.0 ± 0.0 abc	34.0 ± 0.0 d	0.1 ± 0.0 ef	74 ± 0.0 abcdefgh
6	PAS4	37.0 ± 0.0 bc	28.0 ± 0.0 h	2.5 ± 0.0 b	65 ± 0.0 ghi
7	PAS5	46.9 ± 0.0 ab	31.0 ± 0.0 f	0.2 ± 0.0 ef	78 ± 0.0 abcd
8	PAS6	38.0 ± 0.0 abc	27.6 ± 0.1 h	0.1 ± 0.0 ef	66 ± 0.1 fghi
9	MKN1	35.9 ± 0.0 c	29.0 ± 0.0 g	0.2 ± 0.2 ef	65 ± 0.0 hi
10	MKN2	48.2 ± 0.2 a	35.4 ± 0.2 c	0.9 ± 0.0 c	84 ± 0.3 a
11	BFG	43.2 ± 0.2 abc	26.6 ± 0.3 i	0.6 ± 0.0 d	70 ± 0.4 cdefgh
12	CMF	44.7 ± 0.1 abc	32.0 ± 0.0 e	0.8 ± 0.0 cd	77 ± 0.1 abcde
13	CSD	23.9 ± 9.0 d	33.7 ± 0.1 d	0.7 ± 0.0 cd	76 ± 0.6 i
14	PAG	48.6 ± 0.1 a	35.0 ± 0.0 c	0.3 ± 0.0 e	84 ± 0.0 a
15	SWMF	38.9 ± 0.0 abc	29.0 ± 0.0 g	0.8 ± 0.0 cd	68 ± 0.1 defghi
16	BMF	42.0 ± 0.0 abc	34.0 ± 0.0 d	0.6 ± 0.0 d	76 ± 0.0 abcdef
17	FMF	44.0 ± 0.0 abc	31.8 ± 0.0 e	0.9 ± 0.0 c	76 ± 0.0 abcdefg
18	AMF	39.7 ± 0.1 abc	32.2 ± 0.2 e	3.0 ± 0.0 a	72 ± 0.3 bcdefgh
19	CTE	38.2 ± 0.2 abc	28.0 ± 0.0 h	0.8 ± 0.0 cd	66 ± 0.2 efghi
20	KSD	38.5 ± 0.0 abc	35.0 ± 0.0 c	0.0 ± 0.0 f	73 ± 0.0 abcdefgh
Mean		39.2 ± 2.3	31.8 ± 0.8	0.7 ± 0.2	72 ± 2.8

Means with the same letters are not significantly different from each other (*p* < 0.05, Tukey’s test). Codex Alimentarius Standard (fructose: 31–42%, glucose: 23–32%, sucrose: ≤5%, reducing sugar: ≥60%) [9,10].

**Table 8:** Comparison among sugar contents of local and imported honey in Saudi Arabia.

Physicochemical Properties (Sugar Content)	Local Honey (Mean ± SE)	Imported Honey (Mean ± SE)	*p*-Value
Fructose (%)	39.7 ± 0.6	39.2 ± 2.3	0.833
Glucose (%)	31.5 ± 0.7	31.8 ± 0.8	0.780
Sucrose (%)	2.8 ± 0.9	0.7 ± 0.2	0.020 *
Reducing sugar (%)	71.2 ± 1.3	72.0 ± 2.8	0.078

Asterisk represents a significant difference between local and imported honey for each single attribute (*p* < 0.05, *t*-test). The mean percentages of fructose, glucose, sucrose, and reducing sugar of local and imported honey were within the permitted limits (fructose: 31–42%, glucose: 23–32%, sucrose: ≤5%, reducing sugar: ≥60%) of the International Codex Alimentarius and GSO standard [9,10].

## Data Availability

Data is contained within the article.
